# Implications of dominance deviations for reproductive and morphological traits in the genetic management of the Pura Raza Española horse

**DOI:** 10.1186/s12711-026-01075-4

**Published:** 2026-08-05

**Authors:** Davinia I. Perdomo-González, Mercedes Valera, Antonio Molina, Pedro Azor, Juan P. Sánchez

**Affiliations:** 1 Dpto. de Producción Animal, Facultad de Veterinaria, Universidad Complutense de Madrid, Madrid, Spain; 2 Dpto. Agronomía, ETSIA, Universidad de Sevilla, Ctra. Utrera Km, 41005 Seville, Spain; 3 Dpto. Genética, Universidad de Córdoba, Ctra. Madrid-Córdoba Km 396, 14071 Córdoba, Spain; 4 Real Asociación Nacional de Criadores de Caballos de Pura Raza Española (ANCCE), 41012 Seville, Spain

## Abstract

**Background:**

Dominance is a non-additive genetic effect involving allele interaction at the same locus. In animal breeding, it has often been ignored due to its complexity and the low accuracy of pedigree-based estimates. This study analysed dominance variance components for reproductive traits [age at first foaling (AFF), reproductive efficiency (RE), interval between first and second foaling (I12)] and morphological traits [scapulo-ischial length (SIL), dorsal sternal diameter (DSD), knee circumference (KC)] in Pura Raza Española (PRE) mares. The aim was to assess the magnitude of dominance effects across traits to determine whether alternative models are required for breeding value estimation or if dominance deviation predictions should be reconsidered in strategies such as mating design.

**Results:**

Mares with full sisters and at least one foaling were identified and additional records of other females foaling in the same stud and year were also selected. The number of mares with reproductive and morphologic records was 3967 and the pedigree included 11,331 individuals with an average relatedness coefficient of 5%. First, condensed probabilities of identity based on pedigree data were estimated using a recursive algorithm. Next, a linear model in which variance components (dominance, additive and their covariance) were estimated, using the pedigree and phenotypic data. The dominance variance was partitioned into a term linked to relationships between non-inbred individuals and another linked to relationships between inbred individuals, with the latter assumed to be correlated with the additive effect. The broad-sense heritability values oscillated between 0.64 (SIL) and 0.20 (RE) while the narrow-sense heritability ranged between 0.05 (I12) and 0.39 (KC) and estimates of the additive-dominance correlation varied between − 0.43 (RE and I12) and − 0.69 (DSD). The consideration of dominance deviations in mating design would allow a relative increase of 0.05% (DSD) to 3.4% (I12) in the phenotypic performances of newborns foals.

**Conclusions:**

Dominance deviations represent a relevant genetic phenomenon in the PRE breeding programme. If ignored, they may bias additive variance estimates and lead to an overestimation of expected selection response. Models that include dominance effects could be valuable for designing mating strategies aimed at maximising the overall genetic value and phenotypic performance of newborn foals.

**Supplementary Information:**

The online version contains supplementary material available at 10.1186/s12711-026-01075-4.

## Background

The concept of dominance deviation is used to describe the extent to which the phenotype of the heterozygote deviates from the expected intermediate value of the two homozygotes [1]. In the absence of dominance, the breeding values (A) and genotypic values (G) are identical; nevertheless, when examining a single locus, there is usually a difference between the G and the A of a genotype, which is referred to as the dominance deviation (D) [2]. Statistically, dominance deviation represents interactions between alleles at the same locus. However, the concept of dominance has largely been overlooked in animal breeding applications, even though it has been shown to play a key role in production traits of livestock species over the last 60 years or more [3–5]. The reasons for ignoring dominance are mainly due to the complexity of the statistical models required and the low accuracy of estimates obtained using pedigree-based models, since such models require records from full-sib relationships, which occur less frequently in many species, including horses. Dominance relationships across individuals were presented many decades ago [6, 7] but the first efficient computational method used to analyse dominance was published in 1990 [8]. Later, a comprehensive review and description of pedigree-based covariance between relatives in the presence of dominance gene action was presented by De Boer and Hoeschele [9]. These authors showed how, in a population experiencing inbreeding, covariances between breeding values and dominance deviations arise as the departure from the Hardy–Weinberg equilibrium increases, giving rise to four genetic parameters (the additive genetic variance, the variance of fully inbred dominance deviations, the variance of non-inbred dominance deviations, and the covariance between additive genetic effects and fully inbred dominance deviations) instead of the usual two. However, in practice, even when dominance is included using pedigree information, covariances due to inbreeding have largely been ignored [10]. Recently, Fernández et al. [10] developed, using pedigree and phenotypic records, an equivalent model to estimate variance components in inbred lines using the full model described by De Boer and Hoeschele [9]. This equivalent model can be implemented using standard quantitative genetics software and approaches such as Bayesian methods implemented via the Gibbs sampler, and it allows us to estimate dominance variance, in addition to residual and additive variances (as well as the covariance between additive and dominance effects). Therefore, there are very few estimates of genetic parameters which properly consider the covariance between breeding values and dominance deviations in inbred populations, such as dairy cattle [11], sheep [12] and rabbits [10].

The Pura Raza Española (PRE) horse, originating from Spain, holds a significant place in the country's history, cultural identity and traditions, and has also played a crucial role in the Spanish economy [13, 14]. Established in 1912, the PRE studbook operates as a closed breeding program, exclusively registering horses with documented parentage. In the early 1980s, molecular techniques, including paternity tests, were introduced, providing a comprehensive pedigree spanning over 40 years [15]. As of 2024, there are 290,407 active PRE horses spread across 65 countries over five continents, overseen by the Real Asociación Nacional de Criadores de Caballos de Pura Raza Española (ANCCE) [16].

While the PRE horse population is of a considerable size, comprehensive genealogical analyses have revealed strikingly high levels of inbreeding [17–19]. This can be attributed to the limited number of founders, the intense selection carried out for morphofunctional traits, and the practice of inbred matings within studs seeking to maximize the influence of emblematic ancestors of the breed. The PRE Breeding Program, launched in 2003 to improve conformation and functionality [20], has often placed less enphasis on fertility. However, fertility -shaped by factors such as genetics, age, nutrition, and management practices [21, 22], is essential for maintaining a sustainable equine population. Ensuring reproductive success is therefore critical for both population resilience and economic efficiency, making the careful management of mare reproductive performance a key priority.

The objective of this paper was to estimate genetic parameters for morphological and reproductive efficiency traits in an equine breed, while accounting for inbreeding and dominance deviations, an approach that, to our knowledge, has not been previously applied. Specifically, additive and dominance variances, as well as the covariance between additive and dominance effects were estimated in Pura Raza Española mares. Incorporating dominance deviation models may provide a valuable tool for mating design by helping to maximize the overall genetic value of offspring, and we therefore also explore the implications of including such information in mating decisions.

## Methods

### Phenotypic records

Reproductive traits, age at first foaling in months (AFF), reproductive efficiency (RE, %) and interval between first and second foaling in months (I12), were calculated, following the methodology outlined by Perdomo-González et al. [23], from records of over 360,000 horses provided by the ANCCE. Reproductive efficiency (RE) was defined as the ratio of the total number of foalings to the optimal number of foalings a mare could have throughout her entire life. The optimal foaling number was calculated individually for each mare, considering the age at which she first foaled and, in the case of premature culling from the breeding herd or the end of her productive life, the age at her last foaling. An optimal foaling frequency of one per year was assumed, starting from the initial foaling. To ensure the accuracy of the data, mares born after 1970 (the inception year of genetic parentage analysis in the PRE) that had produced their first foal in a stud specializing in foal production, characterized by an output of over 12 foals per year were selectively chosen. To refine the selection criteria further, mares engaged in leisure or sport activities were excluded. The final criteria included mares which had produced their first foal between 4 and 7 years old, with an interval between the first and second foaling no greater than 5 years, an average interval between foalings greater than 9 months, and an interval between the last and penultimate foalings below 5 years. Finally, mares with full sisters, at least one foaling and records of other females foaling in the same stud and year were identified for the analysis. Trait distributions were evaluated using Shapiro–Wilk tests and graphical inspection. Although some variables showed slight deviations from normality, departures were generally moderate, and no data transformation was applied. Final data base comprised 3967 mares, 1388 of which had between 1 and 6 full sisters also selected. Then a pedigree of 11,331 animals (8897 females and 2434 males) was generated, and the population structure analysis was carried out using ENDOG v4.8 software [24].

In addition, the morphology of the selected mares was also analysed focusing on three morphological traits: scapulo-ischial length (SIL), dorsal-sternal diameter (DSD) and knee circumference (KC). The SIL is the straight-line distance from the shoulder joint to the point of the buttock, the DSD is the circumference of the thorax taken from the top of the withers via the sternum, and the KC is the circumference of the knee joint. Biometric traits were meticulously gathered during the official breed controls, which are mandatory for all PRE horses before inclusion in the official studbook. Trained inspectors used standard measuring sticks and non-elastic measuring tape, and all the measurements were taken from the left side of the horse, while it stood on a stable, flat surface, maintaining a natural position, following the procedures outlined by Sánchez-Guerrero et al. [20].

### Model for analysis

First, condensed probabilities of identity based on pedigree data were estimated using a recursive algorithm following the method of Karigl [25]. Calculations were performed using an in-house Fortran software, VRR (Variance of Realized Relationships) incorporating the hashing procedures implemented in the BLUPF90 package [26, 27] to store previously computed coefficients, as described in Fernández et al. [10]. Then a linear model was implemented fitting dominance and additive genetic effects, so that the variance components (dominance, additive and their covariance) were estimated from pedigree and phenotypic data. To analyse the three morphological and three reproductive efficiency traits in PRE mares, the univariate models described by Fernández et al. [10] were implemented:

#### Full-dominance model

1 $$ {\mathbf{y}} = {\mathbf{X}}{\boldsymbol{\upbeta }} + {\mathbf{Tt}} + {\mathbf{Ws}} + {\mathbf{f}}{\mathrm{b}} + { }\left( {\mathbf{Z}} \; 0 \right)\left( {\begin{array}{*{20}c} {\mathbf{a}} \\ {{\mathbf{a}}^{*} } \\ \end{array} } \right) + \left( 0 \; {\mathbf{Z}} \right)\left( {\begin{array}{*{20}c} {{\mathbf{d}}_{{\mathbf{i}}}^{*} } \\ {{\mathbf{d}}_{{\mathbf{i}}} } \\ \end{array} } \right) + 	{\mathbf{Zd}}_{{\mathbf{R}}} + {\mathbf{e}}, $$

where **y** is the vector of observations, **β** is the vector of fixed effects, **t** the vector of the mare’s first foaling year, **s** is the vector of the mare’s first foaling stud, **f** is a vector with inbreeding coefficients, b is the assumed linear effect of inbreeding on the trait, **e** is the vector of residuals, and **a**, **d**_**i**_, and **d**_**R**_ are additive, “dominance in inbreeding”, and “dominance in random mating” genetic effects, respectively. **X** **T**, **W**, and **Z** are incidence matrices relating observations to the corresponding effects. Following the formulation of de Boer and Hoeschele [9], inbreeding depression is modeled separately from dominance deviations. Thus, adjusting for inbreeding depression does not remove dominance effects but accounts for the change in the genotypic mean associated with inbreeding, allowing dominance deviations to represent the residual dominance variation around this adjusted mean. The effects **t** and **s** are factors for which the following covariance matrices were assumed: Var(**t**) = **I**$\sigma_{t}^{2}$ and Var(**s**) = **I**$\sigma_{s}^{2}$, where I denotes an identity matrix of appropiate dimension. The covariance structure of the genetic effects is expressed using the Kronecker product as:

$$\begin{aligned} &Var \left( {\begin{array}{*{20}c} {\begin{array}{*{20}c} {\boldsymbol{a}} \\ {{\boldsymbol{a}}^{\user2{*}} } \\ \end{array} } \\ {\begin{array}{*{20}c} {{\boldsymbol{d}}_{{\boldsymbol{i}}}^{\user2{*}} } \\ {{\boldsymbol{d}}_{{\boldsymbol{i}}} } \\ \end{array} } \\ \end{array} } \right) = {\boldsymbol{G}}_{0} \otimes \left( {\begin{array}{*{20}c} {\boldsymbol{A}} & {\boldsymbol{C}} \\ {\boldsymbol{C}}^{\prime} & {{\boldsymbol{D}}_{{\boldsymbol{i}}} } \\ \end{array} } \right), \\ &{\mathrm{where}}\; \, {\boldsymbol{G}}_{0} = \left( {\begin{array}{*{20}c} {\sigma_{a}^{2} } & {\sigma_{{a,d_{i} }} } \\ {\sigma_{{a,d_{i} }} } & {\sigma_{{d_{i} }}^{2} } \\ \end{array} } \right)\end{aligned}$$

Matrices **A**, **D**_**i**_, and **C** represent, respectively, additive relationships, dominance relationships under inbreeding, and additive-dominance relationships. Effects ${\boldsymbol{a}}^{\user2{*}}$ and ${\boldsymbol{d}}_{{\boldsymbol{i}}}^{\user2{*}}$ must be added to facilitate calculations, but it is important to note that these sub-vectors do not have any associated phenotypes; they only serve to create the right covariance structure so that the Kronecker product can be applied, as described by Fernández et al. [10]. Summary statistics of the additive, dominance, and additive–dominance relationship matrices were computed to describe the structure of the pedigree information used to estimate dominance covariance components. The results are provided in Additional file 1 Table S1.

More specifically, **β** included the geographical area (3 levels; Spain, rest of Europe and rest of the world), the mare’s year of birth grouped in 10-year intervals (5 levels; before 1976, between 1976 and 1985, between 1986 and 1995, between 1996 and 2005 and between 2006 and 2015), herd size in the year of the mare’s first foaling (14 levels) and genetic origin related to the coat color (3 levels; chestnut, of Arab origin; grey, of North-African origin; and black and brown, of European origin) as systematic effects. The mare’s first foaling year (**t**) and the first foal stud (**s**) were fitted as random effects, assuming independent normal prior distributions.

#### Partial dominance model

2 $$ {\mathbf{y}} = {\mathbf{X}}{\boldsymbol{\upbeta }} + {\mathbf{Tt}} + {\mathbf{Ws}} + {\mathbf{f}}{\mathrm{b}} + { }{\mathbf{Za}} + { }{\mathbf{Zd}}_{{\mathbf{R}}} + {\mathbf{e}}, $$

In this model, as in the previous, the covariance matrix of ${\boldsymbol{d}}_{{\boldsymbol{R }}}$ is ${\boldsymbol{D}}_{R} \sigma_{{d_{R} }}^{2}$, and elements of ${\boldsymbol{D}}_{R}$ were computed from the probabilities of the nine condensed identity states, as described in Fernández et al. [10].

#### No-dominance model

3 $$ {\mathbf{y}} = {\mathbf{X}}{\boldsymbol{\upbeta }} + {\mathbf{Tt}} + {\mathbf{Ws}} + {\mathbf{f}}{\mathrm{b}} + { }{\mathbf{Za}} + {\mathbf{e}}, $$

For which all the terms have been already described.

Variance components were estimated using Bayesian methods (Gibbs sampling using flat priors) with the program GIBBSF90+ [26, 27]. All the models were run for 1,000,000 iterations, with a burn-in of 100,000 and a thin interval of 100. Post-Gibbs analysis included an estimation of the effective sample size [28] using CODA [29], and a visual inspection of the traceplots of the chains for the different parameters. As a model-comparison criterion, we computed the Deviance Information Criteria [30].

We also estimated the following population parameters as described by Fernández et al. [10]: additive variance ($\sigma_{a}^{2}$), a component produced by dominance structure with no inbreeding ($\sigma_{{d_{R} }}^{2}$), a component produced by dominance structure with inbreeding ($\sigma_{{d_{i} }}^{2}$), a component produced by the covariance of additive and dominance effects in the presence of inbreeding ($\sigma_{{a,d_{i} }}$), the narrow (additive) and broad-sense (additive + dominance) heritability values (h^2^ and H^2^, respectively), the proportion of phenotypic variance attributed to dominance variation (D^2^), the additive-dominance correlation ($r_{{a,d_{i} }}$) and the residual term variance ($\sigma_{e}^{2}$). Additive heritability denotes the proportion of a trait’s variation that is due to additive genetic effects. Dominance heritability represents the additional contribution to trait variation arising from dominance effects, which are interactions between alleles at the same locus, beyond what is captured by additive heritability. In addition, to compare the variance components estimated in these models, we implemented the original definitions from Legarra [31] across inbreeding levels ranging from 0 to 0.38 (the maximum inbreeding level observed in the studied population). This approach allows reporting meaningful estimates of genetic parameters for populations characterized by different levels of inbreeding.

To assess whether including dominance effects in the model would influence mating decisions—under an assortative mating design—as well as the selection of future stallions or mares, we performed an additional breeding values prediction using the previously estimated genetic parameters. In this analysis, we held out records from 118 mares born between 2010 and 2014, all of which had no offspring and had known sire and dam. In this scenario without own phenotypes, these 118 mares represent putative matings between a set of sires and dams, allowing the comparison of predicted genetic values across the different models. This comparison evaluated the consequences of including dominance deviations in the genetic evaluation model at the time of mating design.

Thus, for each one of these 118 mares (i.e., representing 118 putative matings between their respective parents), three genetic value predictions were obtained. Based on those predicted genetic values for each mare, the top 50 matings were identified. The genetic value predictions were computed by accounting for inbreeding depression and by including the corresponding genetic terms of each model. For example, in the full dominance model, the predicted genetic value was calculated as the sum of the additive genetic value, the dominance effect under inbred mating, and the dominance effect under non-inbred mating. From this sum, the individual inbreeding depression was subtracted. This value was computed as the inbreeding coefficient of the mating multiplied by the estimated inbreeding depression parameter (i.e., the regression coefficient of the phenotype on the inbreeding coefficient included in the model).

We then reported both the percentage of coincidence among the top 50 matings across models and the actual average phenotypic performance of the selected matings (i.e., the average phenotype of the 50 top mares, whose records were not included in the evaluation). Finally, to assess the impact of mating design on final selection decisions, we assumed that only 20% of the putative offspring would be retained as future mares (10 out of the 50 realized matings). In this step, the percentage of coincidence among the top 10 animals was evaluated using additive value predictions (EBV) from the three models, as this criterion reflects the expected transmitting ability of the candidates.

## Results

Based on the selected mares, a pedigree with 11,331 animals was reconstructed and the population structure was analysed (Table 1). The pedigree completeness level of the selected mares was over 99% for the first four generations and the mean complete, mean equivalent complete and mean maximum generations were 3.7, 5.9 and 9.4, respectively. In addition, the pedigree completeness level of the reconstructed pedigree remained over 70% up to the 4th generation and the mean complete, mean equivalent complete and mean maximum generations were 2.8, 4.7 and 7.8, respectively. While the number of founders was 717, the number of ancestors was 683 and the number of effective founders and effective ancestors were 36 and 32, respectively; the number of ancestors accounting for 50% of the current genetic diversity was 13. This relatively low number of founders and ancestors is reflected in the mean inbreeding coefficient (4.5%) and the average relatedness coefficient (5.1%) for the animals in the pedigree, while these values were even higher for the selected mares (5.3% and 5.5%, respectively). Finally, the number of unique sire-dam combinations (i.e., each mating that produced offspring) in the pedigree was 8660. Of these, 1283 combinations produced more than one offspring, and a total of 3237 animals had at least one full sibling (sharing both sire and dam). The selected mares belonged to a total of 3056 families, of which 477 sire-dam combinations produced more than one daughter, and a total of 1388 mares had at least one full sister (sharing both sire and dam).

**Table 1:** Genealogical population parameters

Parameters	Pedigree	Selected mares
Number of animals	11,331	3967
Pedigree completeness level at 2nd generation	0.918	1
Pedigree completeness level at 3rd generation	0.855	0.999
Pedigree completeness level at 4th generation	0.736	0.999
Mean complete generations	2.813	3.693
Mean equivalent complete generations	4.655	5.878
Mean maximum generations	7.575	9.373
Number of founders	717	
Number of ancestors	683	
Effective number of founders	36	
Effective number of ancestors	32	
Number of ancestors accounting for 50% of the genetic diversity	13	
Mean inbreeding coefficient	0.045	0.053
Minimum inbreeding coefficient	0.000	0.000
Maximum inbreeding coefficient	0.384	0.369
Average relatedness coefficient	0.051	0.055
Number of couples	8660	3056
Number of couples with more than one son/daughter	1283	477
Number of animals with at least one complete sibling	3237	1388

The number of selected mares with phenotypic records ranged from 3967 (AFF and RE), to 3092 (SIL), as shown in Table 2. The average phenotypic values for the reproductive traits were 56.7 and 17.1 months for AFF and I12, respectively, while the average value for RE was 46.9%. The reproductive trait with the highest coefficient of variation was I12, with 49.3%. The average values for the morphological traits were 160.1, 74.2 and 31.9 cm (cm) for SIL, DSD and KC, respectively, and the highest coefficient of variation was found for DSD (5.3%).

**Table 2:** Descriptive statistics for the reproductive and morphological traits analysed in the Pura Raza Española mares

	Number	Mean (SE)	Min	Max	CV (%)
AFF (months)	3967	56.70 (0.152)	45.00	84.00	16.84
RE (%)	3967	46.89 (0.289)	5.88	100.00	38.75
I12 (months)	3576	17.06 (0.141)	10.00	60.00	49.28
SIL (cm)	3092	160.10 (0.096)	139.00	187.00	3.32
DSD (cm)	3170	74.24 (0.069)	42.00	96.00	5.27
KC (cm)	3096	31.89 (0.025)	21.00	45.00	4.40

*AFF* age at first foaling in months, *RE* reproductive efficiency expressed as %, *I12* interval between first and second foaling in months, *SIL* scapulo-ischial length in centimetres, *KC* knee circumference in centimetres, *DSD* dorsal sternal diameter in centimetres, *SE* standard error, *CV* coefficient of variation

Posterior mean estimates of variance components are provided in Table 3 for reproductive traits and in Table 4 for morphological traits. DIC comparisons revealed that, for all the traits analysed, the model that showed the best fit with the data (the lowest DIC values) was the full dominance model. Also, for all the traits, the estimated mare’s first foaling year (${\sigma}_{t}^{2}$), the first foaling stud (${\sigma}_{s}^{2}$), and the additive (${\sigma}_{a}^{2}$) variances were similar between the models. In general, residual variances (${\sigma}_{e}^{2}$) were lower in the full dominance model compared to the partial dominance and no dominance models.

**Table 3:** Estimates of additive and dominance variance components and the additive–dominance covariance for the reproductive traits in Pura Raza Española mares

	${\sigma}_{t}^{2}$	${\sigma}_{s}^{2}$	${\sigma}_{a}^{2}$	${\sigma}_{a,{d}_{i}}$	${\sigma}_{{d}_{i}}^{2}$	${\sigma}_{{d}_{R}}^{2}$	${\sigma}_{e}^{2}$	${r}_{a,{d}_{i}}$	DIC
AFF									
Full dominance									27,454.5580
Mean	4.64	15.79	17.56	− 24.54	59.47	6.28	51.69	− 0.63	
SD	2.04	2.38	4.23	14.07	55.86	5.62	5.20	0.11	
ESS	5800.00	5305.39	177.60	23.66	12.16	73.44	124.04	25.08	
Partial dominance									28,158.7426
Mean	4.64	15.86	13.95			9.11	52.86		
SD	2.02	2.39	3.50			6.33	5.36		
ESS	8900.00	8900.00	429.99			167.89	245.51		
No dominance									28,307.6082
Mean	4.71	15.90	16.30				59.21		
SD	2.07	2.36	3.32				2.89		
ESS	7546.18	7997.69	1031.26				1492.29		
RE									
Full dominance									27,644.4695
Mean	7.80	37.23	38.31	− 32.41	153.61	20.83	219.88	− 0.43	
SD	4.02	6.54	11.92	44.43	172.95	19.05	18.89	0.39	
ESS	6860.00	6860.00	184.13	26.82	15.33	75.56	100.47	11.21	
Partial dominance									33,570.9405
Mean	7.77	37.21	32.31			26.66	224.03		
SD	4.03	6.47	10.01			20.14	18.08		
ESS	8000.00	7051.99	418.30			142.30	215.36		
No dominance									33,656.7627
Mean	7.78	37.55	36.55				244.52		
SD	4.12	6.58	9.76				9.64		
ESS	7739.11	7460.58	607.45				1001.88		
I12									
Full dominance									23,828.3631
Mean	0.35	1.89	3.07	− 13.62	198.37	4.12	52.78	− 0.43	
SD	0.30	0.84	2.41	14.80	71.66	4.26	5.05	0.34	
ESS	6662.09	4694.98	17.84	11.67	33.15	74.49	101.64	10.45	
Partial dominance									25,263.3061
Mean	0.37	1.96	3.34			5.16	60.19		
SD	0.31	0.87	2.06			4.15	4.14		
ESS	7739.27	2331.90	104.98			129.61	176.12		
No dominance									25,302.73678
Mean	0.38	2.03	3.91				64.36		
SD	0.31	0.87	2.01				2.29		
ESS	8000.00	2786.92	174.98				389.27		

Posterior means and standard deviations (SD), effective sample size (ESS) of variance components, effect of inbreeding and Deviance Information Criterion, in the three models. *AFF* age at first foaling, *RE* reproductive efficiency, *I12* interval between first and second foal, ${\sigma}_{t}^{2}$ mare’s first foaling year variance, ${\sigma}_{s}^{2}$ first foaling stud variance, ${\sigma}_{a}^{2}$ additive genetic variance, ${\sigma}_{a,{d}_{i}}$ covariance between additive genetic variance and fully inbred dominance deviations, ${\sigma}_{{d}_{i}}^{2}$ fully inbred dominance deviations variance, ${\sigma}_{{d}_{R}}^{2}$ non-inbred dominance deviations variance, ${\sigma}_{e}^{2}$ residual variance, ${r}_{a,{d}_{i}}$ correlation between additive genetic variance and fully inbred dominance deviations, *DIC* Deviance Information Criteria

**Table 4:** Estimates of additive and dominance variance components and the additive–dominance covariance for the morphological traits in Pura Raza Española mares

	${\sigma}_{t}^{2}$	${\sigma}_{s}^{2}$	${\sigma}_{a}^{2}$	${\sigma}_{a,{d}_{i}}$	${\sigma}_{{d}_{i}}^{2}$	${\sigma}_{{d}_{R}}^{2}$	${\sigma}_{e}^{2}$	${r}_{a,{d}_{i}}$	DIC
SIL									
Full dominance									14,838.5021
Mean	0.24	3.62	10.10	− 12.35	20.52	7.94	6.11	− 0.60	
SD	0.17	0.70	1.85	6.06	11.53	2.99	2.13	0.18	
ESS	4242.04	3855.14	229.74	18.38	15.43	134.37	170.82	13.42	
Partial dominance									15,405.2638
Mean	0.25	3.65	8.32			10.18	5.49		
SD	0.18	0.69	1.66			3.29	2.42		
ESS	7320.22	6854.32	664.43			251.77	322.41		
No dominance									17,871.6854
Mean	0.25	3.72	11.25				12.44		
SD	0.18	0.72	1.46				1.08		
ESS	7003.21	6675.57	2594.90				2907.42		
DSD									
Full dominance									6041.9953
Mean	0.30	3.89	4.64	− 5.69	7.60	1.78	5.39	− 0.69	
SD	0.15	0.57	0.81	1.36	2.66	1.24	1.06	0.06	
ESS	5120.00	5120.00	495.30	83.82	32.67	92.30	140.09	21.29	
Partial dominance									17,594.1641
Mean	0.30	3.91	3.85			2.24	5.51		
SD	0.16	0.59	0.75			1.38	1.12		
ESS	8000.00	8000.00	617.37			160.97	206.70		
No dominance									18,246.3110
Mean	0.30	3.95	4.34				7.14		
SD	0.15	0.59	0.71				0.55		
ESS	8000.00	8000.00	1649.94				2010.54		
KC									
Full dominance									7913.1267
Mean	0.02	0.23	0.75	− 0.90	1.42	0.46	0.59	− 0.62	
SD	0.01	0.05	0.14	0.37	0.75	0.26	0.20	0.14	
ESS	6481.67	4284.44	232.35	26.70	26.47	100.13	143.77	19.85	
Partial dominance									8259.62567
Mean	0.02	0.23	0.63			0.60	0.55		
SD	0.01	0.05	0.12			0.26	0.20		
ESS	7477.91	6466.28	619.45			199.02	243.86		
No dominance									9949.7354
Mean	0.02	0.22	0.77				0.99		
SD	0.01	0.05	0.11				0.08		
ESS	7304.60	7133.86	2177.89				2591.19		

Posterior means and standard deviations (SD), effective sample size (ESS) of variance components, effect of inbreeding and Deviance Information Criterion, in the three models. *SIL* scapulo-ischial length, *KC* knee circumference, *DSD* dorsal sternal diameter, ${\sigma}_{t}^{2}$ mare’s first foaling year variance, ${\sigma}_{s}^{2}$ first foaling stud variance, ${\sigma}_{a}^{2}$ additive genetic variance, ${\sigma}_{a,{d}_{i}}$ covariance between additive genetic variance and fully inbred dominance deviations, ${\sigma}_{{d}_{i}}^{2}$ fully inbred dominance deviations variance, ${\sigma}_{{d}_{R}}^{2}$ non-inbred dominance deviations variance, ${\sigma}_{e}^{2}$ residual variance, ${r}_{a,{d}_{i}}$ correlation between additive genetic variance and fully inbred dominance deviations, *DIC* Deviance Information Criteria

In the full dominance model of reproductive traits (Table 3), the additive variance ($\sigma_{a}^{2}$) values were 3.07 (I12), 17.56 (AFF) and 38.31 (RE). These values were intermediate between both dominance with random structure variance ($\sigma_{{d_{R} }}^{2}$, 6.28 and 20.83) and dominance with inbred structure variances ($\sigma_{{d_{i} }}^{2}$, 59.47 and 153.61) for AFF and RE, respectively. For I12, the $\sigma_{{d_{R} }}^{2}$(4.12) and $\sigma_{{d_{i} }}^{2}$ (198.37) were higher than $\sigma_{a}^{2}$.

In the full dominance model for morphological traits (Table 4), the estimated $\sigma_{a}^{2}$ values were 0.75 (KC), 4.64 (DSD) and 10.10 (SIL), which were higher than the estimates for $\sigma_{{d_{R} }}^{2}$ in the three traits (0.46, 1.78 and 7.94, for KC, DSD and SIL, respectively); $\sigma_{{d_{i} }}^{2}$ estimates were higher than $\sigma_{a}^{2}$ variance for all of them, 1.42, 7.60 and 20.52, for KC, DSD and SIL, respectively.

The covariance between additive genetic effects and fully inbred dominance deviations ($\sigma_{{a,d_{i} }}$) values were negative for all traits, ranged from − 32.41 (RE) to − 0.90 (KC). The correlation $r_{{a,d_{i} }}$, computed as $r_{{a,d_{i} }} = \frac{{\sigma_{{a,d_{i} }} }}{{\sqrt {2*\sigma_{a} *\sigma_{{d_{i} }} } }}$ [10], were negative and of large magnitude (− 0.63, − 0.43, − 0.43, − 0.60, − 0.69 and − 0.62 for AFF, RE, I12, SIL, DSD and KC, respectively). Standard deviations are generally large, mainly due to the relatively small data sets and the low heritability values.

Similarly, narrow-sense heritability (h^2^), broad-sense heritability (H^2^) and dominance ratio (D^2^) posterior mean values are reported in Table 5. As expected, the H^2^ was higher than both h^2^ and D^2^, in all cases. The h^2^ values obtained in the full dominance models for an F value equal to the population mean inbreeding (F = 0.053) were, in general, similar to those obtained in the no dominance model as in RE, I12 and KC (0.12 and 0.11 for RE, 0.05 and 0.06 for I12, 0.39 and 0.38 for KC) while for AFF and DSD the h^2^ values in the full dominance models were slightly higher than the no dominance model (0.19 and 0.17 for AFF, 0.31 and 0.28 for DSD). Only for SIL the no dominance model had a h^2^ value (0.41) higher than in the full dominance model for an F value equal to the population mean inbreeding.

**Table 5:** Posterior mean values (SD) of narrow-sense and broad-sense heritabilities (h^2^, H^2^) and the dominance ratio (D^2^) for reproductive and morphological traits in Pura Raza Española mares

Reproductive traits	Model	h^2^	D^2^	H^2^	Morphological traits	Model	h^2^	D^2^	H^2^
AFF	Full dominance (F = 0)	0.18 (0.043)	0.07 (0.059)	0.25 (0.055)	SIL	Full dominance (F = 0)	0.36 (0.063)	0.28 (0.106)	0.64 (0.082)
	Full dominance (F = 0.053)	0.19 (0.045)	0.09 (0.063)	0.26 (0.057)		Full dominance (F = 0.053)	0.38 (0.061)	0.31 (0.101)	0.64 (0.081)
	Partial dominance	0.14 (0.036)	0.09 (0.065)	0.24 (0.06)		Partial dominance	0.30 (0.058)	0.36 (0.116)	0.66 (0.093)
	No dominance	0.17 (0.034)	–	–		No dominance	0.41 (0.049)	–	–
RE	Full dominance (F = 0)	0.12 (0.036)	0.06 (0.059)	0.18 (0.056)	DSD	Full dominance (F = 0)	0.29 (0.048)	0.11 (0.077)	0.40 (0.073)
	Full dominance (F = 0.053)	0.12 (0.037)	0.08 (0.065)	0.20 (0.058)		Full dominance (F = 0.053)	0.31 (0.050)	0.13 (0.074)	0.40 (0.072)
	Partial dominance	0.10 (0.030)	0.08 (0.061)	0.18 (0.058)		Partial dominance	0.24 (0.046)	0.14 (0.087)	0.39 (0.078)
	No dominance	0.11 (0.029)	–	–		No dominance	0.28 (0.043)	–	–
I12	Full dominance (F = 0)	0.05 (0.038)	0.07 (0.069)	0.12 (0.068)	KC	Full dominance (F = 0)	0.37 (0.063)	0.23 (0.126)	0.59 (0.102)
	Full dominance (F = 0.053)	0.05 (0.035)	0.20 (0.083)	0.23 (0.072)		Full dominance (F = 0.053)	0.39 (0.066)	0.25 (0.122)	0.59 (0.100)
	Partial dominance	0.05 (0.029)	0.07 (0.058)	0.12 (0.060)		Partial dominance	0.31 (0.058)	0.30 (0.128)	0.61 (0.106)
	No dominance	0.06 (0.028)	–	–		No dominance	0.38 (0.049)	–	–

*AFF* age at first foaling, *RE* reproductive efficiency, *I12* interval between first and second foal, *SIL* scapulo-ischial length, *KC* knee circumference, *DSD* dorsal sternal diameter, *h2* narrow sense heritability, *H*^*2*^ broad-sense heritability,* D*^*2*^ dominance ratio

The dominance ratios ranged from 0.08 (RE) to 0.31 (SIL) for the full dominance model for an F value equal to the analysed population mean value, and between 0.07 (I12) and 0.36 (SIL) for the partial dominance model.

**Fig. 1:**
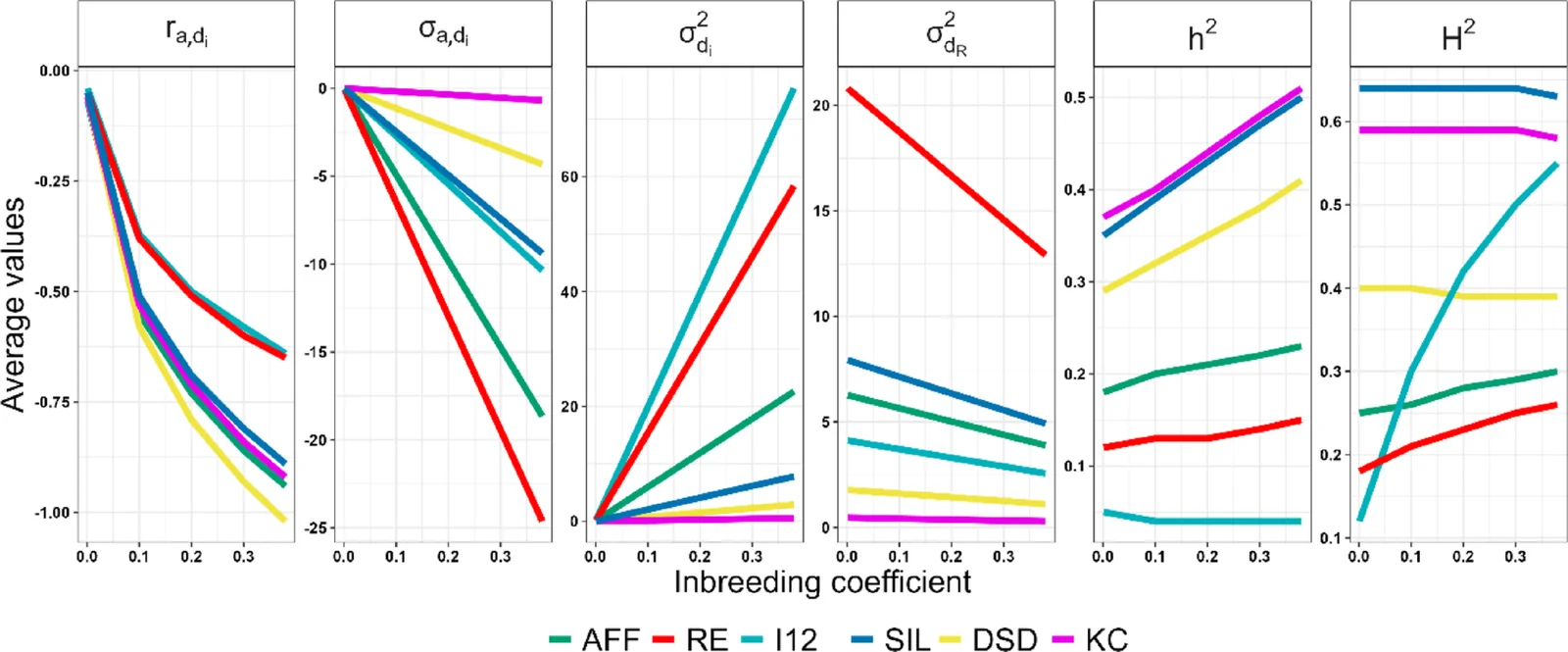
Variation of genetic variance components and genetic parameters across inbreeding levels (F = 0–0.38). *AFF* age at first foaling in months, *RE* reproductive efficiency as %, *I12* interval between first and second foaling in months, *SIL* scapulo-ischial length, *KC* knee circumference, *DSD* dorsal sternal diameter, ${r}_{a,{d}_{i}}$ correlation between additive genetic effects and fully inbred dominance deviations, ${\sigma}_{a,{d}_{i}}$ covariance between additive genetic effects and fully inbred dominance deviations, *h2* narrow-sense heritability, *H*^*2*^ broad-sense heritability

To report meaningful estimates of genetic parameters, the estimates across different levels of inbreeding are presented in Fig. 1. For all the analysed traits, the correlation between breeding values and dominance deviations ($r_{{a,d_{i} }}$) was increasingly negative as inbreeding increased. A similar trend was observed for the covariance between additive genetic effects and fully inbred dominance deviations ($\sigma_{{a,d_{i} }}$) as well as for the variance of non-inbred dominance deviations ($\sigma_{{d_{R} }}^{2}$); both also became more negative with increasing inbreeding. In contrast, the variance of fully inbred dominance deviations ($\sigma_{{d_{i} }}^{2}$) and the narrow-sense heritability (h^2^) increased with inbreeding, while the broad-sense heritability (H^2^) remained virtually constant, except for I12, which showed an increase as inbreeding rose.

Table 6 summarizes the degree of concordance between selection models, the mean phenotypic values of the top 50 ranked animals for each model, and the overlap of the top 10 animals (from top 50) according to the additive genetic values. Concordance among the top 50 animals was generally high (> 70%) for most traits, ranging from a minimum of 72% (between partial-dominance and complete dominance models for KC) to a maximum of 94% (between no-dominance and partial-dominance models for RE, between partial-dominance and full-dominance models for SIL and between no-dominance and complete dominance models for DSD and KC). Mean phenotype values of the best 50 animals varied markedly between traits. For morphological traits, differences between models were minor, whereas for reproductive traits, the full-dominance model consistently identified animals with better average phenotypic values compared to the no-dominance model. In fact, the best 50 animals from the complete dominance model had an average phenotype of 3.4% and 2.9% lower than the best 50 animals from the no-dominance model for I12 and AFF, respectively, (for both traits, the lower the better), while for RE an increase of 0.5% was observed. Finally, the overlap within the top 10 animals revealed clear differences between traits in the animal selection according to the additive value, oscillating this overlapping from 10% (between no-dominance and full-dominance model for KC) to 100% (between partial and full-dominance models for SIL). Considering only the overlapping between the no-dominance and the full-dominance models the highest differences were seen for the reproductive traits, especially for I12 (just a 60% of coincident).

**Table 6:** Overlap among top-ranked matings across models and average phenotypic performance

Models	Top 50			Average phenotype			Top 10		
	1–2	1–3	2–3	1	2	3	1–2	1–3	2–3
AFF	92	84	86	60.60	60.16	58.80	80	80	70
RE	94	80	78	50.75	50.50	51.02	90	80	80
I12	86	76	80	15.12	14.30	14.60	70	60	40
SIL	92	92	94	163.68	163.82	163.86	90	100	90
DSD	88	94	92	74.46	74.44	74.50	90	90	90
KC	90	94	72	32.14	32.18	32.16	10	90	20

The table reports the percentage of overlapping animals within the top 50 across models based on predicted genetic values, the average phenotype of the top 50 animals selected by each model, and the percentage of overlapping among the top 10 animals (within the top 50) based on additive values. Models: 1 = no-dominance, 2 = partial-dominance, 3 = full-dominance; models fitted excluding the own phenotypes of the 118 held-out mares. *AFF* age at first foaling in months, *RE* reproductive efficiency in %, *I12* interval between first and second foaling in months, *SIL* scapulo-ischial length in centimetres, *KC* knee circumference in centimetres, *DSD* dorsal sternal diameter in centimetres

## Discussion

There is overwhelming evidence that non-additive effects and environment factors contribute to phenotypic variance in model organisms as well as in plant and animal breeding [32, 33]. According to the classic theory of statistical genetics, dominance deviation is the difference between the observed genotypic value and its expected additive value in the absence of dominance [2]. These deviations represent the effects of pairing of alleles (or gametes) which constitute genotypes, which are not considered when alleles (or gametes) are studied separately. Since the mean effects of the genes and the breeding values of the genotypes are a function of gene frequencies in the population, the dominance deviations also depend on these and are, therefore, population-based properties, and not simply measures of the degree of dominance [2]. Estimation of additive and non-additive variance components relies on the observed phenotypic similarity among relatives and the expected contributions of additive and non-additive effects to that similarity [2, 34]. Non-additive genetic effects are rarely tested, as it is generally assumed that additive models capture most of the locus's contribution to a trait, even under dominance architectures, except in the most extreme cases [35]. However, our recent increased knowledge about biological pathways and gene networks has highlighted the importance of gene–gene interactions. While some authors argue that a substantial fraction of genomic variance in populations may arise from these interactions [36–38], part of this contribution may be statistically absorbed into additive genetic variance; however, this does not diminish the biological relevance of gene–gene interactions nor the importance of explicitly modeling non-additive effects.

This work presents, for the first time in an equine population, estimates of dominance variance components based on condensed probabilities of identity and a series of dominance models, including models accounting for the covariance between breeding values and dominance deviations, for economically relevant traits in the PRE horse. The analysed traits were selected because they capture key aspects of reproductive and morphological performance in horses. AFF and I12 describe the onset and early progression of reproductive life, while RE reflects lifetime reproductive efficiency. In contrast, SIL, KC, and DSD are morphological traits related to conformation and functional performance [39, 40]. Together, these traits represent biologically distinct domains with different expected genetic architectures. Reproductive traits typically show low heritability and have often been hypothesised to be more influenced by non-additive genetic effects, whereas morphological traits are generally considered to be more strongly determined by additive genetic variation and tend to exhibit higher heritability. However, the results of the present study only partially align with these expectations, as dominance-related components were comparatively more pronounced for morphological traits in our data.

The pedigree structure is always a key issue in genetic analyses, as additive variance is estimated based on the resemblance between relative. Comparing the similarity of related and unrelated individuals allows the estimation of additive genetic effects. Information from full siblings further helps to capture non-additive effects, including dominance deviations, because full sibs have a non-null probability of sharing by descendant both alleles in the genotype, not only individual alleles; nonetheless caution should be posed since maternal environmental effects can also largely contribute to the resemblance between this type of sibs, potentially up-biasing the genetic parameter estimates [41]. Even though the pedigree analysed makes up only 3.5% of the total PRE population examined by Perdomo-González et al. [15], there are notable similarities in the population structure. Both populations exhibit high pedigree completeness indices (over 85%) for the 2nd and 3rd generations. Additionally, although the study population has a slightly smaller number of founders and ancestors compared to the total PRE population (1,053 founders and 1,023 ancestors), the number of effective founders is nearly identical (33).

The descriptive statistics revealed that the mean values of the traits we analysed matched results in the latest publications about PRE mares for I12, SIL, DSD and KC. The AFF mean value (56.7) was just slightly lower and the RE mean value (46.9) was 2% higher than that reported by Perdomo-González et al. [42] in a PRE mare population. One study analysing how PRE horse morphology differs between genders [20] showed similar or slightly lower mean values than those reported here for SIL (157.6, 158.5, and 159.3), DSD (72.2, 73.6, and 73.9) and KC (31.0, 31.1, and 31.2) for mares in three different time periods: mares born before 1990, mares born from 1990 to 2001 and mares born from 2002 to 2013, respectively. Similarly, slightly lower mean values were reported by Gomez et al. [22] in adult female PRE horses for scapulo-ischial length (159.60), chest width (40.78; not directly comparable to DSD) and knee circumference (31.27). It is worth mentioning that, even when it is seen to be decreasing, the coefficient of variation of I12 is still close to 50%, which implies that there is still considerable room for improvement in comparison with the other traits under study.

To estimate the dominance variance from the phenotype requires separating the dominance from the additive of the total phenotypic variance. This is done by comparing similarities (phenotypic covariances) between relatives for which dominance contributes to this similarity and relatives for which it does not. In other words, we can separate dominance variance from additive variance by comparing phenotypic covariances between relatives that share both alleles identical by descent (IBD) locus with the phenotypic similarities of individuals which are not expected to share the two alleles for IBD. In fact, estimates of dominance variance in animal breeding are scarce, with the little data which exists primarily focused on dairy cattle, beef cattle, rabbit and pigs [10, 43–46], in traits such as milk yield, conformation, post-weaning gain, litter size and growth traits. These authors reported dominance variance values ranging from 10 to 50% of the total genetic variance; however, while some of them used estimation models similar to the partial-dominance model used in this work [44, 45], others directly ignored inbreeding when constructing a dominance relationship matrix. Fernández et al. [10] estimated the dominance variance for the number of kits weaned by rabbits in different closed lines. They considered the effect of inbreeding, and compared complete, partial and no dominance models. Results reported by these authors were coherent with ours, with the full-dominance models providing the best fit and, for all analysed traits, the additive variance being similar or slightly lower compared with the no-dominance model. In contrast, the residual variance was lowest in the full-dominance model (and tended to decrease from the no-dominance to the partial- and full-dominance models). This finding agrees with the theory that dominance variance is absorbed from the residual term.

Nevertheless, some important differences can be found between traits, and there are clear indications of different modes of gene action among them. The most notable of these was reported for I12 with very low levels of narrow-sense heritability, even for high levels of inbreeding, while the broad-sense heritability is quite high. This implies that I12 is largely not additive, and that the most relevant gene action is dominance. As can be seen in Fig. 1, the most important form of dominance in this trait is due to inbreeding relationships and this is accompanied by a moderate negative additive–dominance correlation (comparable to RE and less negative than in AFF and the morphological traits). Biologically, a negative ${\sigma}_{a,{d}_{i}}$ indicates that genotypes with high additive merit tend to exhibit unfavourable dominance deviations in inbred populations. Consequently, individuals selected based solely on additive genetic values may systematically express reduced phenotypic performance under increased homozygosity. Ignoring dominance in such cases can, therefore, lead to upwardly biased predictions of selections response and an overestimation of expected genetic gain, particularly in populations under inbreeding. This could be a reason for the very low heritability values that are usually reported for this trait. The narrow-sense heritability value found in this work for I12 was similar to those reported by Perdomo-González et al. [23] (0.05) and by Gomez et al. [22] (0.04) for PRE mares, while the highest heritability value for I12 was reported by Perdomo-González et al. [42] (0.10) in a multi-trait animal model without dominance in a selected population of PRE mares.

In contrast, AFF and RE behave differently, with dominance playing a relevant role but where the additive variance is also important. For these traits, the broad-sense heritability and narrow-sense heritability values reveal smaller differences, depending on the inbreeding value. In both cases, inbreeding relationship is the most important form of dominance, as suggested by the larger variance of fully inbred dominance deviations $({\sigma}_{{d}_{i}}^{2})$ compared to non-inbred dominance deviations $({\sigma}_{{d}_{R}}^{2})$ in Table 3 and by the D^2^ values in Table 5. Narrow sense heritability values (0.19 and 0.12, for AFF and RE, respectively) for an F value equal to the analysed population mean value (F = 0.053) were similar to those reported by Perdomo-González et al. [23], who evaluated inbreeding depression load for AFF (0.16) and RE (0.10) in a selected population of PRE mares, and those reported by Gomez et al. [22] for AFF (0.15). Meanwhile, Perdomo-González et al. [42] also reported slightly higher heritability values for AFF (0.21) in a multi-trait animal model without dominance in a selected population of PRE mares. Slight differences were also found comparing values to those reported by Karlau et al. [47] in Criollo Argentino mares for AFF (0.16), and RE (0.11). Heritability of age at first foaling has been analysed in various horse breeds, with values reported by Gomez et al. [22] for Arab (0.20), Anglo-Arab (0.16), and Spanish Sport Horses (0.32) mares, and by Taveira and Dias [48] for Thoroughbred mares (0.38). These differences are likely due to the challenges in modelling fertility using large datasets and quantitative approaches, which are particularly limited for mares, and to direct fertility estimates based on foaling percentage.

The behaviour of the morphological traits was similar to AFF and RE, with additivity explaining an important percentage of the variance but dominance being the major factor. The most relevant form of dominance for SIL, DSD and KC is the one due to inbreeding relationships. The narrow-sense heritability values for the morphological traits were, in general, lower than those previously reported for the PRE horse breed, which implies that the expected response to selection is lower than previously predicted by models that do not account for dominance effects. Sánchez-Guerrero et al. [20] reported heritability values of 0.39, 0.38 and 0.50 for SIL, DSD and KC, respectively, in a population of 12,381 PRE horses, recorded as linear traits in both males and females. Nevertheless, a high result was also found for SIL by Poyato-Bonilla et al. [19] (0.64), who evaluated inbreeding depression load in a PRE population with both males and females. Lower heritability values have also been reported by Perdomo-González et al. [40] for DSD and thoracic perimeter (0.21, 0.41, respectively) in the current population of Pura Raza Menorquina horse, another autochthonous Spanish horse breed, which is genetically very close to the PRE horse [49]. At the population mean inbreeding (F = 0.053) dominance ratios (D^2^) from the full-dominance model were generally lower than both the broad-sense (H^2^) and the narrow-sense (h^2^) heritability values. The exception was I12, for which D^2^ exceeded h^2^ (0.20 vs 0.05) but remained lower than H^2^ (0.23); moreover, the difference (D^2^ − h^2^) increased with inbreeding. This pattern may partly explain why traditional selection has been less effective for I12 than for AFF, and why I12 shows a high coefficient of variation. In quantitative genetics, the expected response to traditional phenotypic selection depends mainly on additive genetic variance (i.e., h^2^), which is very low for I12, whereas a comparatively larger proportion of the genetic variance is due to dominance (D^2^). Because dominance deviations are not transmitted across generations as predictably as additive effects, and because our results indicate that the dominance component for I12 increases with inbreeding, a portion of the observed phenotypic variability may not translate into a proportional response to selection, potentially contributing to the high coefficient of variation for this trait.

Variance components refer to either a completely outbred or a completely inbred population, neither of which exists in practice. For this reason, it is important to report meaningful estimates of genetic parameters for the analysed population and to estimate genetic progress or the covariance between breeding values and dominance deviations [31]. A comparison of the variation of the genetic parameters estimated with these models as a function of the inbreeding levels of the population highlights the relevance of dominance effects in PRE. As can be seen in Fig. 1, in a theoretical population with an average inbreeding of 0.2, the estimates of narrow-sense heritability increase slightly in comparison with the estimates for the base population, and, as a result of both these factors, the total genetic variance does not increase, since the covariance between additive genetic effects and fully inbred dominance deviations is negative.

Finally, in addition to determining a mismatch between real and expected genetic progress in the population, the non-additive effect has the potential to cause rapid phenotypic changes by giving rise to adaptive novelties in small populations undergoing drift. When effective population size is limited, random genetic drift and the associated increase in homozygosity can rapidly change allele frequencies. Because the additive effect of an allele (the average effect of allele substitution) depends on allele frequencies, these drift-driven changes can modify the partitioning of genetic variance and increase the additive component even when the underlying gene action includes dominance (and/or epistasis). Consequently, part of the genetic variation that is expressed as non-additive variance at one set of allele frequencies may contribute to additive variance after drift, potentially allowing faster short-term phenotypic change than would be expected under purely additive assumptions. Thus, random genetic drift can effectively convert non-additive genetic variance into additive genetic variance, increasing the potential for adaptation to local environments instead of limiting it [50]. This fact highlights an additional use for these types of models: the design of matings to maximize the overall genetic value in the offspring (both heritable and non-heritable). On the one hand, the high concordance in the top 50 animals between no dominance and dominance models indicates consistent selection outcomes across dominance assumptions but also suggest model-specific selection patterns for trait such as KC. The minimal differences in selection outcomes between dominance and no-dominance models for SIL and DSD suggest that rankings (or top-animal overlap) are relatively robust to model specification; however, our variance-component estimates and DIC comparisons indicate that non-additive (dominance-related) variance is not negligible, particularly for SIL. Conversely, the greater model-dependent variation seen in reproductive traits, especially in I12, may reflect the contribution of dominance variance, which can be converted into additive variance through genetic drift in small populations, potentially accelerating adaptive change. At the same time, results indicate that breeder’s selection taking into account the dominance effects will, by itself, have positive consequences on the population phenotype, mainly in reproductive traits such as I12 and AFF but also in a less pronounced way in morphological traits. For I12, the phenotype of the 50 best animals was 3.4% lower (which indicates an improvement) compared to what we would obtain with a pairing design based exclusively on additive value, for AFF it was 2.9% lower, and for RE it was 0.5% higher; whereas for SIL, DSD, and KC, the increase is just 0.1%, 0.05%, and 0.06%, respectively. At this point, it is important to note that while lower values indicate better performance for AFF and I12 (whereas higher values indicate better performance for RE), this is not necessarily true for morphological traits such as SIL, DSD, or KC. Although higher values are generally preferred, this depends on the specific trait and the breeder’s selection goals. On the other hand, the absence of substantial differences in the average phenotypes of the top matings for morphological traits, despite considerable changes in the ranking of animals (as reflected by the overlap percentages), even in the presence of notable dominance effects, may be attributed to the inherent difficulty of accurately estimating dominance for these traits. In practice, we recommend using EBV for long-term selection (additive response), and using total genetic value for mating design to deliver immediate phenotypic gain, recognising that the dominance component is not transmitted predictably across generations; in our data, the expected benefit of this approach is greatest for trait with a larger dominance contribution (e.g., I12).

Alternatively, incorporating a dominance model as a preliminary step in breeders’ selection affects the ranking of top animals, particularly for reproductive traits. In our validation setup, where candidates’ own phenotypes were excluded in all models and selection was based primarily on the parental combination, the full-dominance model consistently identifies individuals with superior mean phenotypic values compared to the no dominance model, despite the latter including the phenotype in the evaluation. This indicates results reflect variation in the elite animal identification across models. These findings highlight that while certain traits demonstrate robustness to model choice, others are highly sensitive to the genetic model applied. Therefore, the observed divergences in rankings and overlaps between models are not solely a matter of prediction accuracy but also reflect the potential of dominance-informed selection strategies to influence both short-term gains and long-term adaptive capacity, particularly in small, drift-prone populations. The results of these models could therefore help us to decide which mating pair, among the top mothers and fathers by additive genetic value, would provide the highest expected genotypic value (additive + dominance deviations). This way, the offspring would inherit the best genes and also express the best gene combinations, even though these combinations are certainly not heritable.

Although dominance effects are not routinely included in genetic evaluations of species in which progeny are usually born one at a time, the presence of dominance has been previously reported in livestock populations, including body size traits [44, 45]. Nevertheless, it is well known that increasing model complexity leads to a higher variance of the estimated genetic parameters, as the available information must be partitioned among a larger number of parameters. Consequently, the amount of information per parameter is reduced when compared with simpler additive models, potentially compromising prediction accuracy.

In agreement with previous studies, the inclusion of dominance effects in the model did not result in a clear improvement of additive breeding value predictions, and animal rankings remained essentially unchanged. This suggests that, from a routine genetic evaluation perspective, additive models may still be sufficient. However, dominance effects may still be of interest for specific applications, such as the optimization of mating plans, where simplified dominance models could represent a reasonable compromise between biological realism and model complexity.

Regarding the possible limitations of this work, we did not fit a maternal (common) environmental effect nor genomic (marker-based) relationship matrices. Moreover, accurate estimation of additive-dominance covariance structures requires substantial levels of inbreeding and informative family structures involving dominance (e.g., full-sibs); when such information is limited and common environmental effects are not explicitly modelled, the partitioning of additive and dominance variance may be less reliable. Therefore, the construction of our dataset prioritized increasing the number of full-sibling relationships to improve information on non-additive components. Full-sibs often share similar management and early-life conditions (e.g., rearing, feeding, housing, and training), which can increase their phenotypic similarity and potentially confound the estimation of dominance variance. However, in horses this effect is expected to be less pronounced than in species producing litters, because mares typically give birth to a single foal per gestation and therefore full siblings are not reared contemporaneously. Consequently, although some shared environmental influences among relatives cannot be entirely excluded, the potential confounding between common environmental effects (e.g., maternal or management-related effects) and dominance variance is likely to be more limited in this species. Another evidence of the lack of bias due to confunding full-sib shared environmental effects with dominance covariance can be extracted from the fact that additive genetic parameters, like narrow sense heritability, are aligned with previous estimates reported from datasets not forced to be dominated by full-sibs relationships.

Regarding genomic information, the additive relationship matrix **A** could be replaced by an additive genomic relationship matrix **G**, and dominance under random mating could be approximated using a genomic dominance relationship matrix constructed from SNP markers. In addition, genomic data allow a more direct modelling of dominance by explicitly fitting SNP genotypes in the model and estimating additive and dominance effects at each marker. In such models, additive effects are typically coded using allele counts, while dominance effects capture heterozygous deviations through genotype-based coding, allowing direct estimation of SNP dominance effects and their contribution to genetic variance [51]. However, our full-dominance model additionally separates dominance under random mating (D_R_), dominance in the presence of inbreeding (D_i_), and includes an additive-dominance covariance structure (*C*), which are defined in terms of identity-by-descent (IBD) probabilities in inbred populations. Reproducing this same partition in a purely marker-based framework would therefore require robust inference of autozygosity or IBD segments (e.g., via ROH or haplotype-based IBD methods), sufficiently dense genotypes, broad genotyping coverage, and careful implementation and validation to guaranteed equivalence with pedigree-IBD-based matrices [52]. It is important to note that genomic relationships do not automatically account for shared environment of full-sibs. Therefore, in a strictly genomic framework, explicit random effects for maternal or common environment effects would also need to be included. Future work should therefore consider the joint modelling of genomic information, SNP dominance effects, and maternal/common environmental effects to strengthen the inference of non-additive components.

## Conclusions

In summary, we have, to our knowledge, estimated for the first time in an equine breed the variance components due to dominance deviations in both reproductive and morphological traits from phenotypic and pedigree information. According to our results, incorporating dominance effects into genetic evaluation models offers valuable refinement beyond additive genetic models, particularly for reproductive traits. These models not only improve the ranking and identification of elite animals but also provide practical guidance for breeding decisions. Specifically, the results can help determine which mating pairs, among top parents selected by additive genetic value, will produce the highest overall genetic value (additive + dominance deviations, accounting for inbreeding depression). This approach ensures that offspring inherit superior genes and express optimal gene combinations, even though such combinations themselves are not heritable. While some traits demonstrate robustness to the choice of genetic model, others are highly sensitive to the inclusion of dominance effects, underscoring their biological relevance. Moreover, dominance-informed selection strategies have the potential to enhance short-term realised performance and long-term adaptive capacity by preserving genetic diversity. Therefore, integrating dominance effects represents a promising strategy to optimize breeding programmes and improve population sustainability, and could significantly impact the Pura Raza Española breeding programme.

## Supplementary Information

Below is the link to the electronic supplementary material.

Additional file 1 Table S1. Summary statistics of the additive (A), dominance with random structure (DR), dominance with inbred structure (Di) and additive–dominance relationship (C) matrices.

## Data Availability

The dataset supporting the results of this study was supplied by the Royal National Association of Pura Raza Española horse breeders (ANCCE). The datasets generated and/or analysed during the current study are available in the Zenodo repository, https://zenodo.org/records/13842975.
