# Supplementary material for: Implications of dominance deviations for reproductive and morphological traits in the genetic management of the Pura Raza Española horse
Source: Genet Sel Evol. 2026 Aug 5;58:59. doi: 10.1186/s12711-026-01075-4 (PMC13501697; doi:10.1186/s12711-026-01075-4)
Supplement: Supplementary file 1 [file 12711_2026_1075_MOESM1_ESM.docx]

| **Matrix** | **Diagonal mean** | **Diagonal SD** | **Q1** | **Median** | **Q3** | **Non-zero off-diagonal (%)** | **Off-diag mean** | **Off-diag SD** | **Q1** | **Median** | **Q3** |
| --- | --- | --- | --- | --- | --- | --- | --- | --- | --- | --- | --- |
| A | 1.045 | 0.051 | 1.010 | 1.029 | 1.063 | 92.144 | 0.051 | 0.043 | 0.024 | 0.044 | 0.067 |
| D_R_ | 0.955 | 0.051 | 0.938 | 0.971 | 0.990 | 73.212 | 0.001 | 0.004 | 0.000 | 4.320e-04 | 0.001 |
| D_i_ | 0.045 | 0.051 | 0.010 | 0.029 | 0.063 | 61.239 | 2.660e-04 | 7.439e-04 | 0.000 | 4.100e-05 | 2.270e-04 |
|  |  |  |  |  |  |  |  |  |  |  |  |
|  | **Non-zero elements (%)** | **Mean** | **SD** | **Q1** | **Median** | **Q3** |  |  |  |  |  |
| C | 76.03333 | 0.002314207 | 0.003764392 | 6.1e-05 | 0.0010985 | 0.0029135 |  |  |  |  |  |

**Additional file 1 Table S1**. Summary statistics of the additive (A), dominance with random structure (D_R_), dominance with inbred structure (D_i_) and additive–dominance relationship (C) matrices.

For A and D_R_ and D_i_, statistics are reported separately for diagonal elements and off-diagonal elements (including the percentage of non-zero off-diagonals); for C, statistics are computed over all elements. Q1 and Q3 denote the 25th and 75th percentiles, respectively.
